# VM-Unet enhanced with multi-scale pyramid feature extraction for segmentation of tibiofemoral joint tissues from knee MRI

**DOI:** 10.1371/journal.pone.0330740

**Published:** 2025-08-28

**Authors:** Xin Wang, Yupeng Fu, Huimin Lu, Yuchen Xia, Xiaodong Cai

**Affiliations:** 1 College of Computer Science and Engineering, Changchun University of Technology, Changchun, China; 2 Information Department, Jilin Qianwei Hospital, Changchun, China; Shijiazhuang Tiedao University, CHINA

## Abstract

In medical imaging diagnosis, accurate segmentation of the knee joint can help doctors better observe and diagnose lesions, thereby improving diagnostic accuracy and treatment effectiveness. Vision Mamba mainly relies on the State Space Model (SSM) for feature modeling, which excels at capturing global contextual information but cannot capture local texture features. Moreover, features of different scales are not effectively integrated, resulting in the model’s weak segmentation ability on small-scale tissues (such as cartilage areas). To this end, this study proposed a novel multi-scale Vision Mamba Unet (VM-Unet) framework named MSPF-VM-Unet to perform the segmentation on the femur, tibia, femoral cartilage, and tibial cartilage in knee MRI images. The proposed MSPF-VM-Unet extends VM-Unet by introducing a designed multi-scale pyramid feature extraction network named MPSK, which synergizes multi-resolution feature extraction with channel-space attention. MPSK network enhances multi-scale local feature extraction through Selective Kernel (SK) convolution and pyramid pooling. The network merges the overall context information extracted by the Vision Mamba encoder to achieve the coordinated optimization of a multi-scale hierarchical feature fusion mechanism and global long-range dependency modeling. The results of the comparative experiments on the OAI-ZIB dataset indicate that MSPF-VM-Unet significantly improves the boundary accuracy and regional consistency of the MRI tibiofemoral joint tissue structure.

## 1. Introduction

Knee arthritis is a chronic disease with a prevalence of 22.9% in people over 40 years old. The prevalence increases with obesity and age [1]. Accurate diagnosis and treatment planning of knee joint diseases are highly dependent on the fine segmentation of knee joint tissues (such as the femur, tibia, and corresponding cartilage tissue). The deep learning-based knee joint MRI image segmentation method has become the mainstream method [2]. Prasoon et al. [3] introduced a tri-planar Convolutional Neural Network (CNN) architecture for tibial cartilage segmentation, where three 2D networks were independently applied to the axial, sagittal, and coronal planes of 3D MRI volumes. They were individually linked to the xy, yz, and zx planes within the 3D image. This algorithm outperformed the mainstream approach by utilizing 3D multi-scale features. To address 3D segmentation challenges, Archit et al. [4] developed a volumetric fully convolutional network ‘μ-Net’ with hybrid loss functions, pioneering automated cartilage segmentation using 3D CNN. This is the first automated cartilage segmentation approach utilizing 3D CNN. Waqas et al. [5] proposed the PSU-Net network model, which is a refinement of the U-Net. The Squeeze and Excitation block with residual connection is introduced to effectively learn the features of the femur, tibia, and patella. Felfeliyan [6] achieved accurate segmentation of MRI bone and cartilage in a small dataset by improving Mask RCNN. Daydar [7] proposed the Multi-resolution Attentive U-Net (MtRA-Unet) for automatic segmentation regarding the femur, tibia, and cartilage of both.

Although deep learning methods have brought breakthroughs in knee MRI image segmentation, in practical applications, due to the complex anatomical structure, fuzzy tissue boundaries, and susceptibility to imaging noise, traditional segmentation models often face challenges such as insufficient multi-scale feature fusion, low efficiency of global context modeling, and limited boundary segmentation accuracy [8]. For example, while U-Net and its variants have advanced considerably in the field of medical image segmentation [9,10], there are still problems, such as the loss of local details and blurred boundaries in the segmentation effect, when dealing with fine-grained segmentation tasks in the multi-tissue interlaced area of the knee joint. In this context, emerging model architectures such as Vision Mamba [11] provide new perspectives for medical image segmentation. Unlike the local receptive field mechanism of traditional CNN, Vision Mamba models the long-range spatial dependencies in images through a parameterized two-dimensional SSM. The Vision Mamba architecture combines the topological structure of U-Net to construct VM-Unet [12], which achieves efficient capture of long-range spatial dependencies. VM-Unet employs pure VSS modules to construct a medical image segmentation model, which has the advantage of capturing global contextual information. However, this design has limitations: local details, such as boundaries, are prone to loss, small lesions are easily overlooked, and there is a lack of multi-scale feature fusion mechanisms, resulting in insufficient interaction between low-level details and high-level semantic information.

To address these issues, this study built upon the VM-Unet architecture and designed a new multi-scale VM-Unet framework named MSPF-VM-Unet to segment the femur, tibia, and cartilage of both in knee MRI images. The core contributions of this paper are: (1) VM-Unet is introduced for the first time in joint tissue segmentation from knee MRI images. A new MRI knee joint tissue segmentation network model, MSPF-VM-Unet, was designed based on the VM-Unet network framework, which realizes the cross-level alignment of global information and local details to make comprehensive use of information at different scales for enhancing the segmentation accuracy; (2) Based on pyramid pooling combined with SK channel attention mechanism, a multi-scale local feature extraction network MPSK was constructed. This model significantly improves the model’s ability to capture multi-scale features of knee joint tissue by fusing cross-layer feature pyramid with channel-space dual-path attention; (3) The Efficient Channel Attention (ECA)channel attention mechanism was incorporated into the skip connection to improve the perception of fine tissue details. The comparative experimental results on the OAI-ZIB public dataset show that in the segmentation tasks of the four key tissues, the average Dice Similarity Coefficient (DSC) is improved by 2.5% in comparison with the baseline model, and the reduction in the Average Hausdorff Distance (HD) amounts to 0.473mm.

## 2. Method

### 2.1. Main architecture of the segmentation network

**Fig 1:**
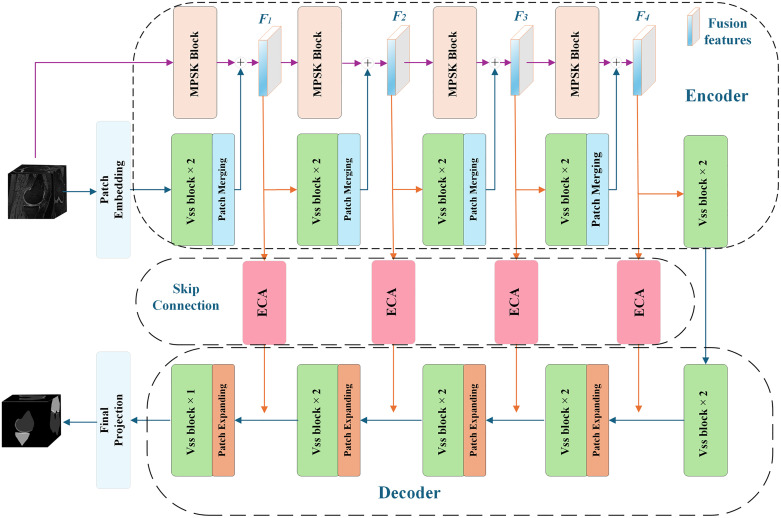
Framework of MSPF-VM-Unet segmentation network model.

The multi-scale feature fusion segmentation network MSPF-VM-Unet is shown in Fig 1, which consists of an encoder, a decoder, and skip connection paths. The encoder structure consists of parallel feature extraction branches formed by the multi-scale pyramid feature extraction network (MPSK-Net) and the visual state space block (VSS) in VM-Unet: the former is responsible for extracting multi-scale local features, while the latter focuses on global contextual features. After fusion, the two types of features form the core structure of the encoder, where F1, F2, F3, and F4 represent the feature maps after fusion at each level, respectively. An ECA channel attention module is inserted into the skip connection path of the encoder and decoder to achieve multi-scale cross-level information fusion. The decoder setting is the same as VM-Unet. Finally, the decoder restores the feature map and the final segmentation result is obtained.

### 2.2. Multi-scale pyramid feature extraction network

#### 2.2.1. Architectural details.

**Fig 2:**
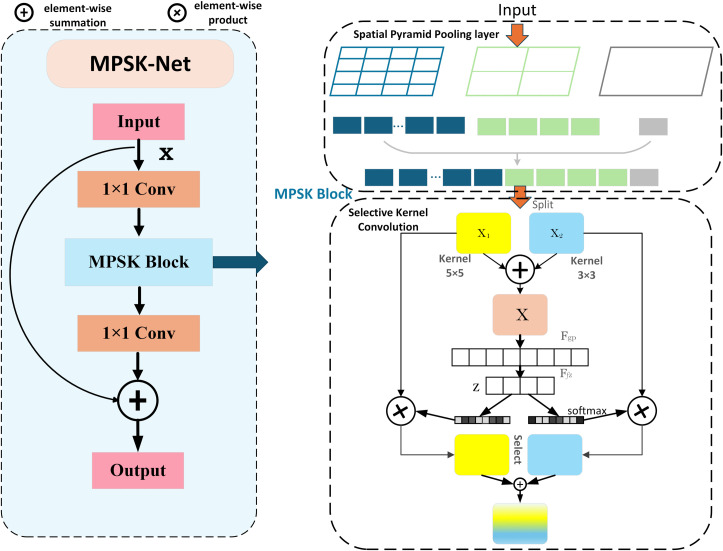
Multi-scale pyramid feature extraction network MPSK.

The encoder of Vision Mamba is better at capturing global context features, but it is not good at capturing multi-scale local information features. Therefore, we design a new multi-scale local feature extraction network MPSK relying on the residual structure of ResNet [13], which is illustrated in Fig 2. Different from the traditional residual network, we replace the 3 × 3 standard convolution kernel with a pyramid pooling module combined with the SK attention mechanism [14]. The global average pooling (GAP) layer and fully connected layer are taken away from the module to better suit the segmentation tasks requiring high-resolution feature maps. MPSK enhances the network’s ability to capture local features at various scales by employing multi-scale feature fusion and an adaptive attention mechanism, thereby more effectively meeting the dual needs of segmentation tasks for local details and global contextual information. When traditional single-scale methods process knee joint MRI images, it is difficult to take into account the details of the tiny cartilage structure and the global anatomical structure of the entire knee joint at the same time, resulting in limited segmentation accuracy [15]. In contrast, the multi-scale pyramid attention mechanism in this study is able to accurately capture features at different scales and achieve more efficient local and global information fusion by using different-sized convolutional kernels and pyramid pooling modules in parallel. The 1 × 1 convolutional kernel, for example, has a small receptive field and can focus on local details to accurately capture the fine texture of the cartilage surface; the receptive field of the 3 × 3 and 5 × 5 convolution kernels gradually increase, which can obtain features in a wider area and help grasp the overall structure of the knee joint tissue. The pyramid pooling module, on the other hand, achieves comprehensive capture of different-scale features in knee MRI images by pooling operations.

#### 2.2.2. Pyramid pooling.

The specific operation process of the pyramid pooling module is as follows: given an input feature map F_in_, the network first performs multi-scale processing on the input feature map. By using various sizes of convolution kernels 1 × 1, 3 × 3, and 5 × 5 in parallel to extract feature information of different scales, feature maps of multiple scales are generated. Specifically, the convolution operations are denoted by C_1 × 1_, C_3 × 3_, C_5 × 5_ respectively, then we have:


$$F_{s1}=C_{1\times 1}\left(F_{in}\right),\;where\;\;F_{s1}\;\in \;{\mathbb{R}}^{H\times W\times C}$$ (1)

$$F_{s\text{2}}=C_{\text{3}\times \text{3}}\left(F_{in}\right),where\;\;F_{s\text{2}}\in \;{\mathbb{R}}^{H\times W\times C}$$ (2)

$$F_{s3}=C_{\text{5}\times \text{5}}\left(F_{in}\right),where\;F_{s3}\in \;\;{\mathbb{R}}^{H\times W\times C}$$ (3)

Next, these feature maps of varying scales are concatenated to obtain the fused feature map. The number of channels of the fused feature map is then restored through a 1 × 1 convolution to obtain, thereby lowering computational complexity and keeping the consistency of the feature dimension. The output feature map of pyramid pooling is upsampled and concatenated with the initial feature map, thereby enriching the spatial representation of the feature map.

#### 2.2.3. SK attention mechanism.

In the MPSK network, the SK attention mechanism is brought in to adaptively select features of different scales. First, the channel splitting operation is carried out to obtain the branching results X_1_ and X_2_. Then, the branching results are fused through element-wise summation to obtain X. The global feature vector of the channel dimension is acquired for X through the global average pooling (GAP) operation. Subsequently, two fully connected layers are used to generate the attention weights z of the corresponding feature maps. These weights are normalized by the Softmax function. Finally, the final output feature map (*F*_*out*_) is obtained by weighted summation of the feature maps *F*_*s1*_, *F*_*s2*,_ and *F*_*s3*_ using these weights. In order to retain the original feature information, the output of MPSK is residually connected to the input feature map (*F*_*in*_) to get the ultimate output feature map. In Fig 2, *F*_*gp*_ represents the global average pooling operation, and *F*_*fz*_ represents the fully connected operation of first reducing the dimensionality and then increasing the dimensionality.


$$F_{MPSK}=F_{in}\;+\;F_{out}$$ (4)

Through the above design, the MPSK module can effectively fuse multi-scale features and adaptively weight features of different scales by the SK attention mechanism, thereby enhancing the network performance in the segmentation task. This design not only improves the model’s ability to capture local details but also enriches feature representation for segmentation tasks through the utilization of multi-scale feature fusion and attention mechanism.

### 2.3. Fusion of global features and local features

In the innovative architecture proposed in this paper, the Vision Mamba encoder is one of the core components. It can capture long-range dependencies in the feature map in the form of a sequence through a unique state transfer mechanism. When dealing with long-range dependencies, traditional CNNs have difficulty capturing long-range feature associations owing to the restricted receptive field of the convolution kernel [16]. Therefore, the multi-scale local features **produced** by MPSK are fused with the global features output by the SSM of Vision Mamba, so that the model can capture the global information of MRI knee joint tissue while maintaining the ability to extract multi-scale local features.

First, the input image is mapped to a high-dimensional feature space through convolutional layers to generate initial features (*F*_*0*_). Then, *F*_*0*_ is flattened into a sequence form, $F_{seq}\in \;{\mathbb{R}}^{N\times D}$and the state space model SSM is used to capture long-range dependencies and output a sequence $Y\in \;\;{\mathbb{R}}^{N\times D}$:


$$\left\{ \begin{array}{c} h_{t}=A{h_{t}}_{-1}+Bx_{t}\\ y_{t}=Ch_{t}+Dx_{t} \end{array}\right.$$ (5)

Then, the sequence is reshaped into a feature map F_1_. By continuously stacking multiple Mamba modules and convolutional layers, higher-level global features can be gradually extracted. At the same time, the MPSK network processes the input image in parallel to extract multi-scale local features (*F*_*MPSK*_).

Finally, the global features *F*_*Mamba*_ output by the Vision Mamba encoder are fused with the multi-scale local features F_MPSK_ extracted by MPSK to generate fused features F_f_. The fused features are then further integrated by the convolution layers to get the ultimate features. Simplify the above process into a unified formula:


$$F_{final}=Conv(Mamba(Conv(X))⊕MPSK(X))$$ (6)

Through this parallel architecture, the Vision Mamba encoder and MPSK network achieve complementary advantages, which enables the model to simultaneously capture global context and multi-scale local information, providing powerful feature extraction capabilities for the knee joint segmentation task.

### 2.4. Skip connections

**Fig 3:**
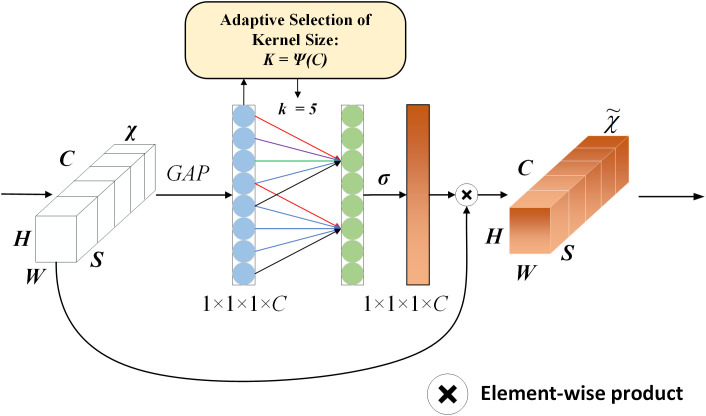
Diagram of 3D efficient channel attention (ECA) module.


VM-Unet still uses the traditional U-Net skip connection approach: the features of the encoder and decoder are simply concatenated without taking into account the importance of features at different scales, which makes it difficult to accurately segment small target tissues. The ECA module [17] is introduced at the skip connection of the architecture in this paper. As shown in Fig 3, the ECA module takes charge of optimizing the feature fusion mechanism of the skip connection to improve the segmentation ability of small target tissues. The traditional U-Net structure directly concatenates the encoder features *X*_*encoder*_ and the decoder features *X*_*decoder*_. The introduction of the ECA mechanism will enhance the channel attention of the encoder feature and optimize the feature transfer process. Specifically, the encoder output features *X*_*encoder*_ are first subjected to GAP operation to extract the global information Z_c_ from the channel:

$$z_{c}=\frac{1}{H\times W}\sum_{i=1}^{H}\sum_{j=1}^{W}X_{encoder}\left(c,i,j\right)$$ (7)

Then, 1D convolution is used to calculate the adaptive weights among channels:


$$\omega_{c}=\sigma \left(Conv1D\left(z_{c}\right)\right)$$ (8)

in which, σ is the Sigmoid activation function. Conv1D(**·**) extracts the correlation among channels through a dynamically adjusted convolution kernel. The obtained attention weight *ω*_*c*_ is re-applied to the original feature map:


$$X_{eca}\left(c,i,j\right)=\omega_{c}\cdot X_{encoder}\left(c,i,j\right)$$ (9)

Finally, the ECA-weighted encoder features X_ECA_ are fused with the decoder features *X*_*decoder*_ by skip connection:


$$F_{skip}=X_{ECA}+X_{decoder}$$ (10)

## 3. Experimental results and discussion

### 3.1. Dataset and evaluation metrics

We evaluated our method using the publicly obtainable OAI-ZIB dataset (https://doi.org/10.12752/4.ATEZ.1.0), which is a knee tissue dataset from the Osteoarthritis Initiative (https://nda.nih.gov/oai/) and includes 507 3D MRI scans. Experts manually annotated the femur, tibia, femoral cartilage, and tibial cartilage. The details of the OAI-ZIB dataset are shown in Table 1. The dataset, covering all Kellgren-Lawrence (KL) grades 0–4, was stratified and split into training, validation, and test sets at a 7:2:1 ratio. Five-fold cross-validation was conducted within the training set to optimize model parameters, with results averaged to reduce bias. The independent validation set was used for final hyperparameter tuning, preventing data leakage. The unseen test set provided an unbiased evaluation of model generalization. This combined stratified splitting and cross-validation strategy ensures robust model performance across diverse data distributions.

**Table 1:** Details of the OAI ZIB dataset.

Parameter	Numerical value
Gender (Male: Female)	262: 245
Age/years	61. 87 ± 9. 33
Osteoarthritis grading	60: 77: 61: 151: 158
(0: 1: 2: 3: 4)	

The DSC and HD are the two commonly used evaluation metrics in medical image segmentation tasks. DSC measures the degree of overlap between the predicted region and the true region, effectively reflecting the overall accuracy of the segmentation. HD measures the maximum distance between boundaries, which can capture the model’s ability to handle boundary details and is particularly suitable for evaluating the segmentation quality of elongated or complex boundary structures such as cartilage. DSC focuses on global consistency in overlapping areas, while HD evaluates the severity of local errors at boundaries. Combining the two metrics allows for a comprehensive evaluation of model performance from both the dimensions of region overlap and boundary accuracy.


$$DSC=\frac{2*TP}{2*TP+FP+FN}$$ (11)

in which TP (True Positive), FP (False Positive), FN (False Negative).

Hausdorff Distance is a measure that describes the degree of similarity between two point sets, A and B.


H(A,B)=max(h(A,B),h(B,A)) (12)

$$h(A,B)=\underset{a\in A}{\max}\left\{\underset{b\in B}{\min}\left\|a-b\right\|\right\}$$ (13)

$$h(B,A)=\underset{b\in B}{\max}\left\{\underset{a\in A}{\min}\left\|b-a\right\|\right\}$$ (14)

### 3.2. Parameter setting and image preprocessing

MSPF-VM-Unet is realized based on PyTorch 2.0.1. We trained on a single RTX 3090 GPU using the AdamW optimizer with a momentum of 0.9. The initial learning rate was set to 0.0001. The training process lasted for 100 epochs, and the batch size was set to 24.

**Fig 4:**
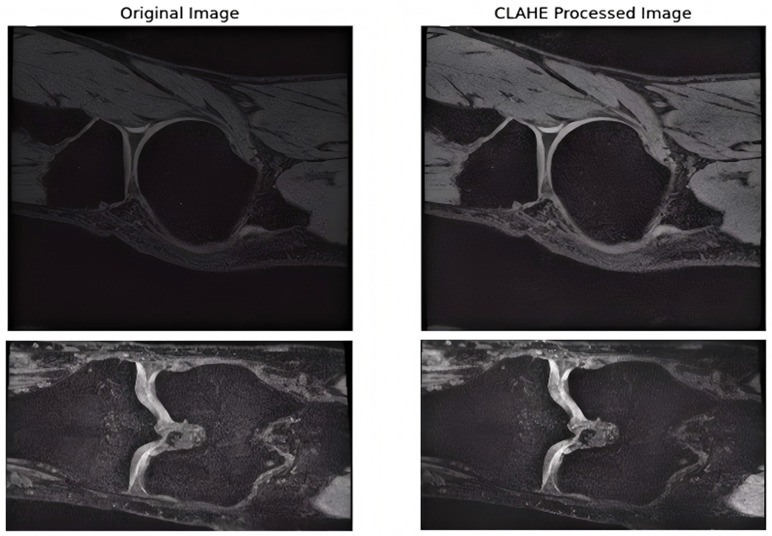
Comparison before and after preprocessing.

Preprocessing is a key step that affects the final segmentation accuracy [18,19]. For MRI images, we started by applying contrast-limited adaptive histogram equalization (CLAHE) to boost the contrast and image details so as to better highlight the tissue structure and boundaries [20]. The image is then standardized using Z-score to remove intensity variations across different scans. Compared with the original images, the processed images exhibit a significant enhancement in contrast. The differences in light and dark between different tissue structures become more pronounced. Meanwhile, the details are much clearer. The previously blurred boundaries become sharp, and the textures are presented more distinctly, which facilitates more accurate segmentation of knee joint tissue. Fig 4 illustrates the contrast diagram of images prior to and following preprocessing.

### 3.3. Experimental results

To evaluate the performance of MSPF-VM-Unet, comparative experiments were conducted with commonly used MRI knee segmentation networks (including recently released networks) on the OAI-ZIB dataset. These models are all based on the encoder-decoder structure, among which nnU-Net has performed well in multiple well-known medical image segmentation tasks [21].

The segmentation results of the femoral bone(FB) and tibial bone(TB) are shown in Table 2. The segmentation results of the femoral cartilage (FC)and tibial cartilage(TC) are shown in Table 3. The numerical values are mean ± standard deviation. The highest DSC and lowest HD achieved for each tissue are in bold. As can be seen in Table 2, our model achieved average DSC of 98.61% and 98.37% for FB and TB segmentation results, respectively. Although the mean DSC of the TB is slightly lower than that of the MtRA-Unet algorithm (98.44%), the mean HD of the TB reached 1.489 mm, which is much lower than that of the MtRA-Unet algorithm (3.31 mm). The mean HD distance of FB is also the smallest among all the compared algorithms. We obtained the same conclusion as in Table 3. In addition, compared to other algorithms, our method achieves smaller standard deviations for most evaluation metrics, indicating the stability of our algorithm. In summary, MSPF-VM-Unet performs well in various knee tissue segmentation tasks.

**Table 2:** Comparison results of femur and tibia segmentation.

Model	FB DSC (%)	HD (mm)	TB DSC (%)	HD (mm)
U-Net [8]	96.43 ± 0.19	6.817 ± 4.17	96.05 ± 0.22	7.335 ± 5.31
nnU-Net [21]	97.87 ± 0.17	2.443 ±2.19	97.17 ± 0.20	3.307 ± 3.21
I-Mask-RCNN [6]	97.60 ± 0.14	4.451 ± 2.12	97.53 ± 0.14	2.989 ± 2.17
MtRA-Unet [7]	98.53 ± 0.15	4.437 ± 1.13	**98.44** **± 0.14**	3.310 ± 1.20
Unet++ [22]	98.39 ± 0.16	4.230 ± 2.33	97.12 ± 0.19	5.931 ± 2.24
V-Net [23]	97.04 ± 0.22	3.235 ± 3.23	96.52 ± 0.13	5.267 ± 3.11
VM-Unet [12]	97.35 ± 0.15	1.933 ± 1.18	96.31 ± 0.17	1.932 ± 1.21
Ours	**98.61** **± 0.12**	**1.472** **± 0.40**	98.37 ± 0.15	**1.489** **± 0.43**

**Table 3:** Comparison results of cartilage segmentation.

Model	FC DSC (%)	HD(mm)	TC DSC (%)	HD (mm)
U-Net [8]	84.73 ± 0.39	4.274 ± 3.34	83.21 ± 0.42	5.935 ± 3.27
nnU-Net [21]	88.51 ± 0.32	3.476 ± 2.26	85.49 ± 0.36	6.851 ± 3.24
I-Mask-RCNN [6]	83.32 ± 0.25	4.846 ± 2.18	80.67 ± 0.30	5.138 ± 3.31
MtRA-Unet [7]	89.19 ± 0.21	5.503 ± 2.19	**86.02** **± 0.31**	4.125 ± 2.33
Unet++ [22]	86.51 ± 0.30	3.686 ± 2.21	85.93 ± 0.33	4.366 ± 2.34
V-Net [23]	87.75 ± 0.28	3.504 ± 3.18	84.09 ± 0.28	4.497 ± 4.35
VM-Unet [12]	85.33 ± 0.31	2.140 ± 1.23	83.17 ± 0.28	2.861 ± 1.34
Ours	**89.57** **± 0.27**	**1.832** **± 0.33**	85.63 ± 0.27	**2.184** **± 1.24**

**Fig 5:**
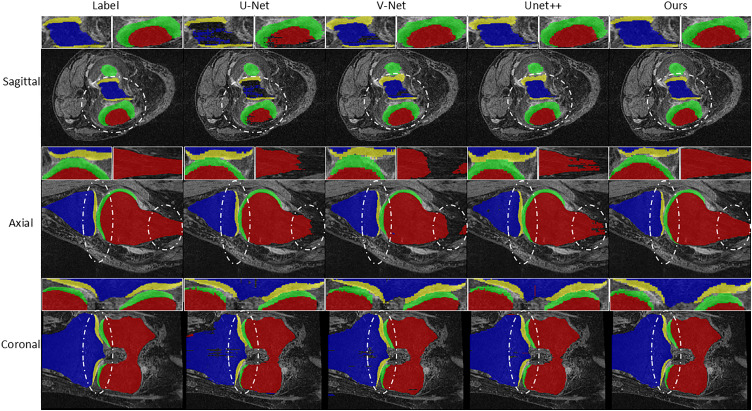
Comparison of segmentation results from the three perspectives.

To better illustrate the segmentation performance of different algorithms, Fig 5 presents the results from three perspectives: sagittal, axial, and coronal. In Fig 5, each row corresponds to the visualization results of the same MRI slice in the same scanning direction by using different segmentation models. Red represents the femur, blue represents the tibia, green represents the femoral cartilage, and yellow represents the tibial cartilage. To compare the performance of different models in terms of detail more intuitively, we marked the key areas with dotted lines in Fig 5 and enlarged them. As can be seen from Fig 5, the tissue contours segmented by MSPF-VM-Unet are smoother, the boundaries are more continuous, and the overall morphology is closer to the ground truth label. In contrast, the segmentation results of the other models have obvious over-segmentation and under-segmentation in multiple areas. For example, at the edge of the femoral cartilage (green area) and the junction of the tibial cartilage (yellow area) with the surrounding tissue, the segmentation results of the comparison algorithm have unclear boundaries and unreasonable connections. While the segmentation results of MSPF-VM-Unet maintain integrity in these key areas, resulting in smoother edges of cartilage tissues without obvious mis-segmentation.

**Fig 6:**
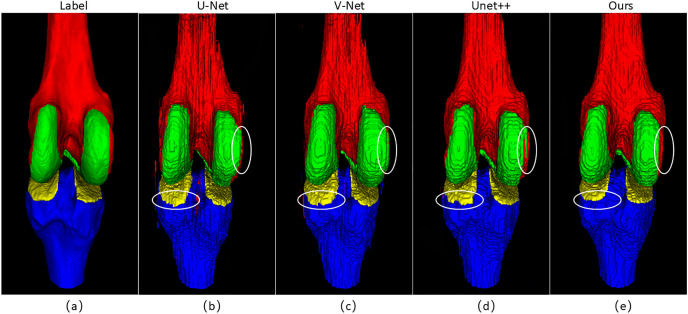
3D visualization effect comparison.

Fig 6 further shows the 3D visualization segmentation results of diverse methods and zooms in on the key area in the upper right corner to highlight the segmentation differences between different models. As can be observed, in the 3D view, the segmentation results of some comparison methods have obvious tissue mis-segmentation, for example, the femoral cartilage appears to be locally missing and connected abnormally. The 3D segmentation results generated by MSPF-VM-Unet are more complete, with clear boundaries and no obvious misprediction areas.

### 3.4. Model performance analysis

A quantitative evaluation of the MSPF-VM-Unet proposed against the baseline model VM-Unet is presented in Table 4. By calculating the paired t-test values (p-value) and 95% confidence interval (CI) on each performance metric, we jointly verify the statistical significance of the performance improvement of MSPF-VM-Unet, as well as its actual magnitude and reliability.

**Table 4:** Statistical comparison of segmentation performance (DSC, HD) between the proposed model and VM-Unet across different anatomical regions.

Region	Metric	Model (mean ± std)	VM-Unet (mean ± std)	95% CI (Model)	p-value
**FB**	DSC	98.61 ± 0.12	97.35 ± 0.15	[98.58, 98.64]	<0.001
	HD	1.472 ± 0.40	1.933 ± 1.18	[1.358, 1.586]	0.01123
**TB**	DSC	98.37 ± 0.15	96.31 ± 0.17	[98.33, 98.41]	<0.001
	HD	1.489 ± 0.43	1.932 ± 1.21	[1.367, 1.611]	0.01763
**FC**	DSC	89.57 ± 0.27	85.33 ± 0.31	[89.49, 89.65]	<0.001
	HD	1.832 ± 0.33	2.14 ± 1.23	[1.738, 1.926]	0.09277
**TC**	DSC	85.63 ± 0.27	83.17 ± 0.28	[85.55, 85.71]	<0.001
	HD	2.184 ± 1.24	2.861 ± 1.34	[1.832, 2.536]	0.01014

In terms of DSC, FB achieved a DSC of 98.61 ± 0.12% (95% CI: [98.58, 98.64]), which is an improvement of 1.30% compared to VM-Unet (p < 0.001). The DSC for TB improved from 96.31 ± 0.17% to 98.37 ± 0.15%, an increase of 2.13% (p < 0.001). A significant improvement (p < 0.001) was recorded for the cartilage structures: the DSC for FC was 89.57 ± 0.27%, an improvement of 4.97% compared to VM-Unet; the DSC for TC was 85.63 ± 0.27%, an increase of 2.96%. The results showed that the model proposed achieved excellent voxel overlap performance in both skeletal structures and soft tissue areas.

For the HD, the value for FB was 1.472 ± 0.40 mm (95% CI: [1.358, 1.586]), significantly lower than the baseline model (a decrease of 23.85%, p = 0.0112), indicating smaller boundary deviations. The HD for TB was 1.489 ± 0.43 mm (95% CI: [1.367, 1.611]), a reduction of approximately 22.95% (p = 0.01763), also achieving statistically significant optimization. In the cartilage region, although the HD of FC decreased to 1.832 ± 0.33 mm (95% CI: [1.738, 1.926]), a decrease of 14.39% compared to the baseline, the difference did not reach statistical significance (p = 0.09277) due to the irregular morphology and low tissue contrast of FC. In contrast, a statistically significant improvement (p = 0.01014) was recorded for the TC, 2.184 ± 1.24 mm (95% CI: [1.832, 2.536]), a reduction of 0.677 mm (23.66%). Quantitatively, MSPF-VM-Unet exhibits superior boundary fidelity.

### 3.5. Ablation experiment

#### 3.5.1. Quantitative analysis.

To verify the effectiveness of each module, we conducted a comprehensive ablation study based on five-fold cross-validation on the OAI-ZIB dataset, with the baseline model being VM-Unet. ECA and MPSK were then added to the baseline model separately. Finally, we incorporated the ECA and MPSK network into the model, leading to the final MSPF-VM-Unet architecture. Table 5 lists the mean values of DSC and HD for the four knee joint tissues (FB, TB, FC, and TC), accompanied by the 95%CI for statistical significance. The numerical values are mean ± standard deviation.

**Table 5:** Ablation experiment results.

Model	Average Dice (%)	95%CI	Average HD (mm)	95%CI
Baseline	90.54 ± 0.23	[90.47, 90.61]	2.217 ± 1.24	[1.865, 2.569]
Baseline + ECA	91.03 ± 0.32	[90.94, 91.12]	2.344 ± 1.32	[1.969, 2.719]
Baseline + MPSK	92.26 ± 0.29	[92.18, 92.34]	1.902 ± 0.83	[1.666, 2.138]
Ours	93.04 ± 0.20	[92.98, 93.10]	1.744 ± 0.62	[1.568, 1.920]

The results in Table 5 show that:(1) introducing ECA alone improved the average DSC from 90.54 ± 0.23% to 91.03 ± 0.32% (95% CI: [90.94, 91.12]), but led to a slight increase of 0.127 mm in HD (95% CI: [1.969, 2.719]). It can be seen that the model does not capture the edge details better. (2) The average DSC of the baseline model equipped with a parallel MPSK is 1.90% higher than that of the baseline model. Meanwhile, the average HD is 1.902 ± 0.83 mm (95% CI: [1.666, 2.138]), a reduction of 0.315 mm (14.21%), verifying the ability of the MPSK module to improve boundary accuracy through multi-scale receptive field coding. (3) The average DSC of the baseline model that combines the ECA attention mechanism with the parallel MPSK network is improved by 2.76%(P < 0.001).In addition, the HD recorded statistically significant (p < 0.05) improvement with 1.744 ± 0.62 mm (95% CI: [1.568, 1.920]), a reduction of 0.473 mm (21.34%). The combined method is more exact in capturing boundary details and further reducing segmentation errors.

#### 3.5.2. Visual comparison.

**Fig 7:**
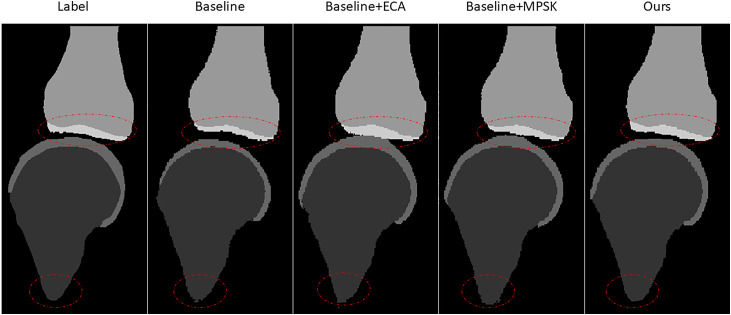
Ablation variant segmentation results.

Fig 7 shows the segmentation results of the four models in Table 5. The Baseline suffers from over-segmentation and contour discontinuities, especially in cartilage areas. ECA improves region homogeneity but shows boundary leakage. MPSK provides a clearer cartilage boundary, but it still lags behind the full model in terms of the uniformity of the internal regions of the tissue and the morphological continuity of the edges. The full model exhibits the best morphology, continuous edges, and superior separation of joint tissues.

#### 3.5.3. Statistical visualization.

**Fig 8:**
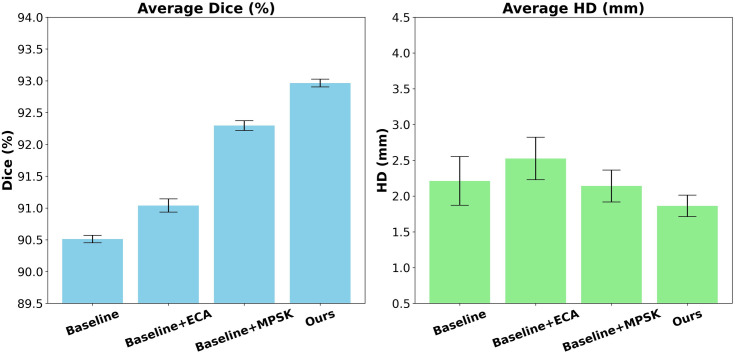
Error-bar plots of the DSC and HD metrics for all variants.

Fig 8 presents error-bar plots of the DSC and HD metrics for all variants across the five folds of the ablation study. Fig 8 can demonstrate the impact of each module on the performance of the proposed model more intuitively. It is clear that the combination of the two modules significantly improved the model performance.

## 4. Discussion

Collectively, the experimental outcomes detailed in Section 3 validate the architectural superiority of our approach. The proposed MSPF-VM-Unet demonstrates outstanding performance in knee MRI tissue segmentation tasks. As detailed in Tables 2 and 3, MSPF-VM-Unet shows varying degrees of improvements in the average DSC compared to other models, except for TB and TC segmentation, where it is slightly inferior to MtRA-Unet (TB: 98.37% vs. 98.44%; TC: 85.63% vs. 86.02%). However, there has been a significant improvement in boundary segmentation accuracy, as evidenced by a significantly lower average HD that far exceeds all competing methods. For example, compared to MtRA-Unet (TB: 1.489 mm vs. 3.31 mm; TC: 2.184 mm vs. 4.125 mm), HD decreased by 55.02% and 47.05%, respectively. This performance gain can be primarily attributed to the synergistic effect of the MPSK module and ECA mechanism. The MPSK module enhances multi-scale local feature extraction through SK and pyramid pooling, and then combines it with the global context information extracted by the Vision Mamba encoder to provide a richer feature representation for segmentation. While the ECA mechanism enhances channel information interaction and optimizes the feature transfer process, enabling the model to capture boundary details more accurately. Ablation studies (seen in Table 5) further quantitatively validated the critical contribution of each component and underscored their importance.

Despite these advancements, two main limitations warrant consideration. Firstly, the model’s computational efficiency needs optimization. As shown in Table 6, compared to the baseline VM-Unet, MSPF-VM-Unet incurs a 23.09% increase in FLOPs (32.62G vs. 26.5G) and a 48.9% longer inference time per volume (70s vs. 47s). This increased computational burden could potentially hinder its deployment in scenarios demanding real-time or near-real-time analysis, such as intra-operative guidance or high-throughput clinical screening. Secondly, the model’s performance on delineating fine, sub-millimeter structures, particularly subtle cartilage fissures or micro-damage at the boundaries, remains an area for improvement. Achieving higher precision in these regions is critical for the early detection of degenerative changes.

**Table 6:** Computational complexity comparison.

Model	Parameters (M)	FLOPs (G)	Processing Time (s)	GPU Memory (GB)
VM-Unet	15.65	26.5	47	19.6
MSPF-VM-Unet	22.67	32.62	70	22.4

To address these limitations and further enhance the framework, future research will focus on:

(1) Implementing model pruning and quantization techniques to significantly reduce computational complexity (FLOPs) and inference time, aiming for clinically feasible deployment.
(2) Developing dedicated boundary refinement modules to specifically boost the segmentation accuracy for subtle structures like cartilage micro-cracks.
(3) Exploring cross-modal learning strategies, potentially incorporating complementary imaging modalities to improve the segmentation fidelity and provide richer structural information for microstructural analysis.

In conclusion, the MSPF-VM-Unet presents a promising approach for accurate and comprehensive segmentation of knee joint tissues from MRI, validated by its superior performance. The integration of MPSK for multi-scale feature extraction and ECA for adaptive feature enhancement proved instrumental in this success. Addressing the identified computational and fine-detail segmentation challenges will be pivotal for translating this method into practical clinical and research tools for osteoarthritis assessment and intervention planning.

## 5. Conclusions

We proposed a new framework, MSPF-VM-Unet, which innovatively integrates multi-scale pyramid feature extraction with VM-Unet to effectively fuse global context information with multi-scale local detail features, significantly improving the segmentation capability of knee joint tissues. The introduction of the ECA attention mechanism further improves the ability to segment small tissue details of the knee joint. The experimental results show that the MSPF-VM-Unet performs excellently in tibiofemoral joint tissues segmentation from Knee MRI. The segmentation accuracy measured as DSC for FB (98.6 ± 0.27%), TB (98.8 ± 0.31%), FC (90.3 ± 2.89%), and TC (86.7 ± 4.07%) is achieved. Simultaneously, the HD metric confirms exceptional boundary fidelity with values significantly outperforming competitors for FB (1.472 ± 0.40 mm), TB (1.489 ± 0.43 mm), FC (1.832 ± 0.33 mm), and TC (2.184 ± 1.24 mm). By delivering anatomically accurate segmentation of knee joint tissues, this study promotes research on knee joint analysis based on quantitative morphology and provides reliable data support for clinical diagnosis and treatment decisions for joint diseases. Future research will focus on computational optimization to achieve a balance between accuracy and efficiency to meet the needs of real-time clinical applications.

## Supporting information

S1 Appendix A comprehensive list of abbreviations used in this paper. (DOCX)
