# Supplementary material for: VM-Unet enhanced with multi-scale pyramid feature extraction for segmentation of tibiofemoral joint tissues from knee MRI
Source: PLoS One. 2025 Aug 28;20(8):e0330740. doi: 10.1371/journal.pone.0330740 (PMC12393787; doi:10.1371/journal.pone.0330740)
Supplement: S1 Appendix — (DOCX) [file pone.0330740.s001.docx]

**Appendix**

| **Abbreviation** | **Full Name** |
| --- | --- |
| VM-Unet | Vision Mamba Unet |
| MSPF-VM-Unet | Multi-Scale Pyramid Feature Extraction Vision Mamba Unet |
| FB | Femur Bone |
| TB | Tibia Bone |
| FC | Femoral Cartilage |
| TC | Tibial Cartilage |
| SSM | State Space Model |
| MPSK | Multi-scale Pyramid Selective Kernel |
| SK | Selective Kernel |
| GAP | Global Average Pooling |
| ECA | Efficient Channel Attention |
| DSC | Dice Similarity Coefficient |
| HD | Hausdorff Distance |
| P-value | Paired t-test value |
| CI | Confidence Interval |
| KL | Kellgren-Lawrence |
| CNN | Convolutional Neural Networks |
| VSS | Visual State Space |
| FLOPs | Floating-Point Operations |

**Table of Abbreviations**
